# Effect of Mild Salinity Stress on the Growth, Fatty Acid and Carotenoid Compositions, and Biological Activities of the Thermal Freshwater Microalgae *Scenedesmus* sp.

**DOI:** 10.3390/biom10111515

**Published:** 2020-11-06

**Authors:** Wiem Elloumi, Ahlem Jebali, Amina Maalej, Mohamed Chamkha, Sami Sayadi

**Affiliations:** 1 Laboratory of Environmental Bioprocesses, Centre of Biotechnology of Sfax, P.O. Box 1177, 3018 Sfax, Tunisia; wiem.elloumi@enis.tn (W.E.); ahlem.jebali@gmail.com (A.J.); maalejamina@yahoo.fr (A.M.); mohamed.chamkha@cbs.rnrt.tn (M.C.); 2 Center for Sustainable Development, College of Arts and Sciences, Qatar University, Doha 2713, Qatar

**Keywords:** *Scenedesmus* sp., growth, pigments, carotenoids, antioxidant activities, enzymatic activities, polyunsaturated fatty acids, cell viability

## Abstract

Carotenoids have strong antioxidant activity as well as therapeutic value. Their production has been induced in algae under stressful culture conditions. However, the extreme culture conditions lead to the Programmed Cell Death (PCD) of algae, which affects their growth and productivity. This study was performed to evaluate the effect of salinity on the physiological and biochemical traits of *Scenedesmus* sp., thermal freshwater microalgae from Northern Tunisia. It was cultured under different NaCl concentrations ranging from 0 to 60 g/L. Results showed a good growth and high contents of total chlorophyll and carotenoids in *Scenedesmus* sp. cultured at 10 g/L of NaCl (salt-stressed 10 (Ss10)). The pigment composition of the Ss10 extract was acquired using HPLC–MS, and showed that the carotenoid fraction is particularly rich in xanthophylls. Moreover, the antioxidant (DPPH and FRAP) and enzymatic inhibition (tyrosinase and elastase) activities of the Ss10 extract were higher compared to those of the control culture. In addition, the cytotoxicity test on B16 cells showed that the Ss10 extract was non-toxic for all tested concentrations below 100 µg/mL. It also showed a rich unsaturated fatty acid (FA) composition. Therefore, these findings suggest that *Scenedesmus* sp. strain cultivated under mild stress salinity could be a source of biomolecules that have potential applications in the nutraceutical and cosmeceutical industries.

## 1. Introduction

High salt concentration is a main limiting factor that affects plant growth and productivity both in aquatic and terrestrial environments [1,2]. Plant cells can mostly tolerate certain changes in salt concentration, through several physiological and molecular processes [3,4]. Biomass composition is influenced by this dynamic change on the metabolic system. It was recently demonstrated that high salt concentration leads to programmed cell death (PCD), which has been considered as a salt response mechanism, in higher plants [5]. The three main types of stress resulting from the high salt concentrations in the culture medium are ionic, osmotic, and oxidative stresses [6]. The first one comes from an imbalance of Na^+^ and K^+^ ionic homeostasis and leads to an inhibition of the enzymes containing K^+^ binding sites by the cation Na^+^ [7]. The osmotic potential of living cells decreases with high salt concentration and therefore leads to a decrease in water amount and a rapid increase in salt concentration inside the cytosol. Moreover, the photosynthetic electron transport is reversibly inactivated after the shrinkage of the intracellular space [8]. Furthermore, stress with NaCl generates an oxidative stress induced by reactive oxygen species (ROS) [9,10]. To avoid damage resulting from salt stress, various antioxidant enzymes are involved [11,12]. Furthermore, compatible metabolites, such as carotenoids and glycerol, produced under salt stress conditions can also serve as antioxidant effects by scavenging free radicals [13] and protect cells against other salt damages [14].

In fact, photosynthetic organisms synthesize terpenoid pigments called carotenoids, which are a family of yellow to orange–red pigments [15]. They include xanthophyll and carotene classes. All xanthophylls, such as antheraxanthin, neoxanthin, violaxanthin, zeaxanthin, and lutein, are produced by higher plants and green microalgae. Actually, all carotenoids directly involved in photosynthesis are called primary carotenoids. However, secondary carotenoids are present in the cells as a response to different environmental factors [16]. Several works have shown that these terpenoid pigments participate significantly in the total antioxidant capacity of microalgae [17,18] and in maintaining or in increasing the possibility of their existence and growth when adverse environmental conditions are applied [16].

A number of studies have shown that low salinity applied during the growth phase of fresh water microalgae contributes to a maximum biomass accumulation [19,20]. However, an increase of NaCl concentrations to 0.4 M inhibits the growth and reduces the quantity of photosynthetic pigments [21]. Furthermore, the effects of NaCl on carotenogenesis were evaluated in different microalgae. The carotenoid accumulation in *Dunaliella salina* and *Dunaliella bardawil* has been studied, and it is well established that it is triggered by high salinity [22,23]. It was found that, at a NaCl concentration of 30%, a significant accumulation of β-carotene occurred in *Dunaliella salina* cells [24]. In *Haematococcus pluvialis* cells, the accumulation of carotenoids was achieved by the addition of 2% NaCl [25]. Further studies showed that the optimum accumulation of carotenoids in *Scenedesmus* sp. was reached under the high salt culture condition of 30 g/L [26], synergistically with high light intensity [27]. Other works showed that *Scenedesmus almeriensis* tolerated well low to medium salinities, between 0 and 5 g/L of NaCl concentrations, giving optimum biomass productivity and lutein content at a salt concentration of 5 g/L, whereas at 3% NaCl the growth was reduced by more than double [21,28].

Many species of green algae can produce valuable secondary metabolites, other than carotenoids, for different uses; examples include polyunsaturated fatty acids (PUFAs), anticancer agents, vitamins, and antiviral drugs. These metabolites are produced when the algae are exposed to stress conditions linked to nutrient deprivation, light intensity, temperature, salinity, and pH. They also have varied applications in health food for human consumption, aquaculture, animal feed, and cosmetics [16,29].

The amount of fatty acids (FAs) in green algae depends on the culture conditions. For example, lower temperatures promote the formation of unsaturated FAs [30,31] and a low pH favors the formation of saturated FAs [32]. Moreover, a high salt concentration leads to a higher ratio of unsaturated FAs in *Dunaliella*, which is a form of adaptation to the high levels of intracellular glycerol formed under high salt concentration [33]. Other studies demonstrated that high salt concentrations can decrease the proportions of unsaturated FAs, resulting in a total decrease in triacylglycerol (TAG) content [34,35].

Secondary carotenoids generated in the chloroplast, under stress conditions, are frequently transported into the cytoplasm to react with FAs and then form carotenoid esters. These hydrophobic molecules accumulate in extraplastidial lipid globules. For example, the overgeneration of astaxanthin in *Haematococcus* induces the accumulation of astaxanthin in oleic acid rich triacylglycerol (TAG) globules in the cytoplasm [36,37]. However, when β-carotene is overproduced, as in *Dunaliella bardawil* cultured under stress conditions, it functions as a secondary carotenoid and accumulates in TAG droplets in the chloroplast [38].

The aim of this work was to determine the impact of low salinity increase on the growth and the fatty acid and carotenoid compositions of *Scenedesmus* sp., and their contribution to the anti-oxidative and anti-enzymatic activities of its extract.

## 2. Materials and Methods

### 2.1. Materials

#### 2.1.1. Chemicals

Acetone was purchased from Acros Organic, Fisher Scientific (Illkirch, France) and used for the extraction. Acetonitrile, methanol grade MS, and Milli-Q water, purchased from Carlo-Erba-SDS (Val de Reuil, France), were used for HPLC-DAD and HPLC–MS analyses. Standards (Sigma-Aldrich, St. Louis, MO, USA) were used to evaluate the biological activities. Reagents used to evaluate the extract activities were as follows: 3-(4,5-dimethylthiazol-2-yl)-2,5-diphenyltetra-zolium bromide (MTT), 1,1-phenyl-2-picrylhydrazyl (DPPH), 2,4,6-tripyridyl-s-triazine (TPTZ), tyrosinase from mushroom, *L*-DOPA substrate, porcine pancreatic elastase, and *N*-succinyl-ala-ala-ala-p-nitroanilide, which were purchased from Sigma-Aldrich. Anhydrous methanolic HCl reagents and boron trifluoride (BF_3_) were of reagent grade.

#### 2.1.2. Cells

Skin melanoma cells (B16) (C57BL/6, Resource No. RBRC-RCB0557) were cultured in Dulbecco’s modified Eagle medium (DMEM) containing 10% fetal bovine serum (FBS) and 1% penicillin/streptomycin solution. They were incubated at 37 °C, under 5% CO_2_ and 95% O_2_ air conditions.

#### 2.1.3. Microalgae

*Scenedesmus* sp. used in the present study was isolated from a thermal freshwater source in Northern Tunisia [39].

### 2.2. Methods

#### 2.2.1. Culture Conditions

The strain was precultured in a 5 L Erlenmeyer flask, under continuous illumination (102 μmol photons m^−2^ s^−1^), and using sterile Modified Detmer’s Medium (MDM) contained in 1 L: 1 g KNO_3_, 0.25 g K_2_HPO_4_, 0.25 g MgSO_4_•7H_2_O, 0.1 g NaCl, 0.01 g CaCl_2_•2H_2_O, 1 mL of Fe solution (200 mg of FeSO_4_·7H_2_O in 100 mL of distilled water), and 1 mL of A_5_ solution [40]. The pH was kept under the optimum range of strain growth (7.80–8.00) by the injection of a pure CO_2_ gas at a flow rate of 0.01 *v/v*/min. The temperature was maintained at 25 °C by controlling the room temperature. Manual agitation was applied to the culture. Furthermore, to study the effect of salt stress, the microalgae was cultured in MDM medium supplemented with 5, 10, 20, 40, and 60 g/L of salt. Briefly, when algal cell population of the preculture *Scenedesmus* sp. reached the exponential growth phase, the biomass was harvested and concentrated in sterile conditions by centrifugation (2000× *g*) for 10 min. Then, it was transferred equally in 250 mL Erlenmeyer flasks, each containing 100 mL of MDM medium added with NaCl. They were incubated at 25 °C with manual agitation. Control culture was also prepared in MDM medium under the same conditions. Each of the salt-stressed and control cultures was prepared in triplicate. In order to preclude contaminations, the medium and the flasks were sterilized in the autoclave at 121 °C for 20 min.

#### 2.2.2. Determination of Microalgae Culture Growth

The cell density of the strain culture was followed daily by recording the optical density (OD) at 760 nm using a UV/VIS Spectrometer (PG instruments, Leicestershire, UK). All analyses were performed in triplicate and the mean value was noted.

#### 2.2.3. Pigment Extraction

Algal culture cells were harvested by centrifugation at 3000× *g* for 10 min. The biomass was first rinsed with distilled water, and then lyophilized. Then, 60 mg of each dried biomass were homogenized for a few seconds (30 s) at 4700 rpm, using a Precellys tissue homogenizer (Bertin Technologies S.A.S, Montigny-le-Bretonneux, France) and acetone as extraction solvent. The supernatant, containing the pigments, was collected after centrifugation at 1000× *g* for 5 min. The extraction procedure was redone three times, and the supernatants were combined and stored at 4 °C for subsequent analysis. This process was carried out in the dark.

#### 2.2.4. Dosage of Pigments

Total chlorophyll and total carotenoid contents were determined according to the Lichtenthaler method [41]. The absorbance of the control and the stressed *Scenedesmus* sp. extracts was measured at 470, 644.8, and 661.6 nm using a UV/VIS Spectrometer (PG instruments, Leicestershire, UK). The total chlorophyll and carotenoid contents were calculated using the equations as follows:

Chlorophyll a (C_a_) (µg/mL) = 11.24 A_661.6_–2.04 A_644.8_ (1)

Chlorophyll b (C_b_) (µg/mL) = 20.13 A_644.8_–4.19 A_661.6_ (2)

Total Carotenoid (C_x+c_) (µg/mL) = [(1000 A_470_)–(1.90 C_a_)–(63.14 C_b_)]/214 (3)

#### 2.2.5. Antioxidant Activity

##### DPPH Scavenging Assay

The radical scavenging capacity of the control and the salt-stressed 10 (Ss10) raw pigment extracts was evaluated as mentioned by Brand-Williams et al. [42]. The scavenging capacity of each sample was compared to ascorbic acid. A 150 µM amount of DPPH solution was freshly prepared by mixing 2.9 mg of DPPH reagent in 50 mL of ethanol. In a 96-well plate, 25 µL of each sample solution were added to 200 µL of DPPH solution. Ascorbic acid (prepared between 0.03 and 15 μg/mL), the control, and the blank of each sample were prepared with the same method and under the same conditions. The plate was then kept in the dark for 60 min. The optical density (OD) was recorded at 517 nm. The scavenging was calculated using Equation (4):

*DPPH* = [(OD_control_ − OD_blank of control_) − (OD_sample_ − OD_blank of sample_)] / (OD_control_ − OD_blank of control_) × 100 (4)

##### Ferric Reducing Antioxidant Power (FRAP) Assay

The FRAP test of the control and the Ss10 raw pigment extracts was evaluated as reported by Benzie and Strain 1996 [43]. This assay showed the ability of the sample to reduce ferric iron (Fe^3+^), which exists in the TPTZ complex, to ferrous iron (Fe^2+^). Briefly, 20 µL of the sample were blended with 230 µL of freshly prepared FRAP reagent. The blank of each sample and the control were also prepared. After incubation for 10 min in the dark, the optical density was recorded at 593 nm. The ascorbic acid was prepared between 2.75 and 176 μg/mL under the same conditions. The reducing potential of iron was calculated using Equation (5):

**%**FRAP = [(OD_control_ − OD_blank of control_) − (OD_sample_ − OD_blank of sample_)] / (OD_control_ − OD_blank of control_) × 100 (5)

#### 2.2.6. Enzymatic Activities

##### Tyrosinase Inhibition Assay

The inhibition of the catalytic action of tyrosinase (oxidation of L-DOPA to dopachrome) was evaluated as described by Masuda et al. 2005 [44]. Briefly, 40 µL of the sample (control and Ss10 raw pigment extracts), 80 µL of sodium phosphate buffer (PBS), and 40 µL of mushroom tyrosinase (92 U/mL) were mixed and incubated in the dark for 10 min. Next, 40 µL of 2.5 mM of L-DOPA were added. The mixture was then incubated for 5 min. The optical density (OD) of the formed dopachrome was recorded at 475 nm. Kojic acid was used as positive control. Experiments were performed in triplicate. The tyrosinase inhibition was calculated using Equation (6):

*(%) Tyrosinase inhibition = (*OD_control_ − OD_sample_) / OD_control_ × 100 (6)

##### Elastase Inhibition Assay

Porcine pancreatic elastase type IV (PPE) inhibition was tested spectrophotometrically, as reported by Kwan et al. 2009 [45], using N-succinyl-ala-ala-ala-p-nitroanilide as substrate. The amount of released p-nitroaniline was determined by measuring the absorbance at 405 nm. Briefly, 70 μL of Trizma-base buffer, 5 μL of elastase, and 10 μL of sample (control and Ss10 raw pigment extracts) were mixed and incubated for 15 min, in the dark. Next, 15 μL of the substrate were added. The mixture was then incubated for 30 min at 37 °C. Quercetin was used as a positive control. Experiments were performed in triplicate. The inhibition percentage of elastase was measured using Equation (7):

*(%) Elastase inhibition = (*OD_control_ − OD_sample_) / OD_control_ × 100 (7)

#### 2.2.7. Cytotoxicity Effect

Cytotoxicity evaluation of B16 cells was carried out using the MTT assay [46]. Different concentrations of the control and the Ss10 raw pigment extracts (2.5 to 100 µg/mL) were applied to the cells. After 24 h of incubation, the medium was changed by 100 µL of a new one and 10 µL of MTT solution. Treated and untreated cells were then incubated at 37 °C for 24 h. Finally, 100 µL of 10% sodium dodecyl sulfate (SDS) were added to disband the formed crystals of formazan. The absorbance was measured at 570 nm using a microplate reader (Thermo Scientific Varioskan Flash, Vantaa, Finland).

#### 2.2.8. Identification of Pigments

High-performance liquid chromatography with diode array detection (HPLC-DAD) (Thermo-Dionex, Les Ulis, France) was performed using a reverse-phase C18 column (MACHERY-NAGEL EC 250 mm/4.5 mm, Nucleodur C18 gravity, 5 µL). The mobile phase was composed of (A) methanol/acetonitrile (60/40, *v/v*) and (B) 10 mM ammonium acetate. The solvent gradient of the mobile phase started from 70% of (A) and increased to 90% over 15 min, then isocratic at 90% for 15 min, 95% of (A) for 5 min, 98% of (A) for 10 min, isocratic at 98% for 10 min, 70% of (A) for 5 min, and isocratic at 60% for 10 min [47]. The used flow rate was 0.7 mL/min. The extract injection volume was 20 μL. The optical density was measured over a range of 210 nm to 700 nm. The peak profile was recorded at 445 nm to facilitate the detection of carotenoids.

The same method was used for the high-performance liquid chromatography–mass spectrometer (HPLC–MS) analyses. They were performed with HPLC-DAD (Thermo Scientific Dionex U3000 (Thermo-Dionex, Les Ulis, France)) online with a quadrupole mass spectrometer (Surveyor MSQ Plus System, Thermo-Dionex, Les Ulis, France). The mass spectrometer (MS) was recorded in positive and negative ionization modes using the operating conditions as follows: ion spray voltage 3 kV, curtain gas 50 psi, Q energy 70 V, cone voltage 50 V, desolvatation temperature 500 °C, and ion energy 0.8 V. In all cases, mass spectra were acquired in the range of 100–1000 Th. Chromeleon^®^, version 6.8 software provided by ThermoScientific Dionex (Les Ulis, France) was used for the treatment of results.

#### 2.2.9. Determination of Fatty Acid Composition

##### Hydrolyses of Microalgae Oil for FAME Conversion

Lipid extraction from the dried biomass of *Scenedesmus* sp. cultured at 10 g/L of NaCl (Ss10) was operated using cyclohexane solvent. The fatty acid composition was determined by analyzing the methyl esters of fatty acids (FAMEs), using GC–MS. A saponification step followed by a methylation catalyzed by BF_3_ (Sap/BF_3_) step were carried out to determine the FAME composition of the sample. Briefly, cyclohexane extract was hydrolyzed with 1 mL of 1M KOH (at 90 °C for 1 h) using a screw-capped glass tube (16.5 × 105 mm). The mixture was acidified with 0.2 mL of 6M HCl. Afterwards, 1 mL of distilled water was added. Then, 1 mL of cyclohexane was added to extract the released free fatty acids (FFAs). Subsequently, the cyclohexane was evaporated and the FFAs were methylated with 1 mL of BF_3_ (at 37 °C for 20 min). The FAMEs were extracted with 1 mL of hexane, which was evaporated on a water bath [48].

##### GC–MS Analyses

FAMEs were analyzed by GC–MS carried out on a Shimadzu Gas chromatograph equipped with a Quadruple Mass spectrometer using electron impact ionization. A DB-17 MS column (60 m length, 0.25 mm diameter) kept at 250 °C was used for the separation of the FAMEs. Cyclohexane solution containing 2 mg of FAMEs was injected in a split ratio of 10:1 applying the following ramp: 180 °C for two minutes, 20 °C per minute from 180 to 280 °C, and hold time for 8 min. The identification of the saturated and unsaturated components was carried by comparing them with the National Institute of Standards and Technology (NIST) profile (2772 SRM).

### 2.3. Statistical Analysis

Results are presented as mean ± standard deviation. Statistical significance was analyzed using *t*-test. *p*-value < 0.05 was considered as statistically significant.

## 3. Results

### 3.1. Cultivation under Stress Conditions

**Figure 1:**
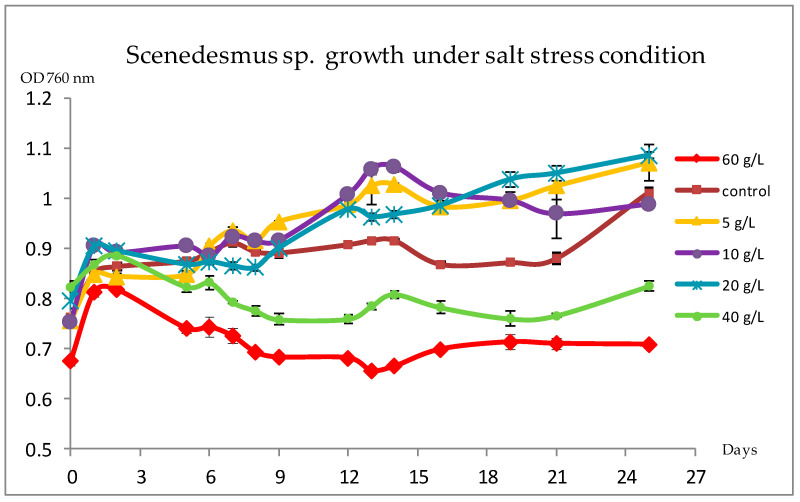
Growth monitoring of *Scenedesmus* sp. cultured in Modified Detmer’s Medium (MDM) (control) and in MDM medium supplemented with 5, 10, 20, 40, and 60 g/L of NaCl, by the measure of the optical density (OD) at 760 nm.

Figure 1 shows that salt concentration has an effect on *Scenedesmus* sp. growth. Indeed, during the first three days, all cultures grown under salt stress have the same growth as the control culture. From the third incubation day, the growth of *Scenedesmus* sp. cultured at 40 and 60 g/L of NaCl declined compared to the control culture (unstressed strain). It then continued to decrease gradually until the last day of measurements. On the contrary, cultures of *Scenedesmus* sp. stressed by 5, 10, and 20 g/L of NaCl showed an increase in the growth compared to the control culture, from the ninth day of measurements. This finding confirmed that *Scenedesmus* sp. can tolerate a certain range of salt concentrations.

### 3.2. Pigment Content of the Stressed Scenedesmus sp.

**Figure 2:**
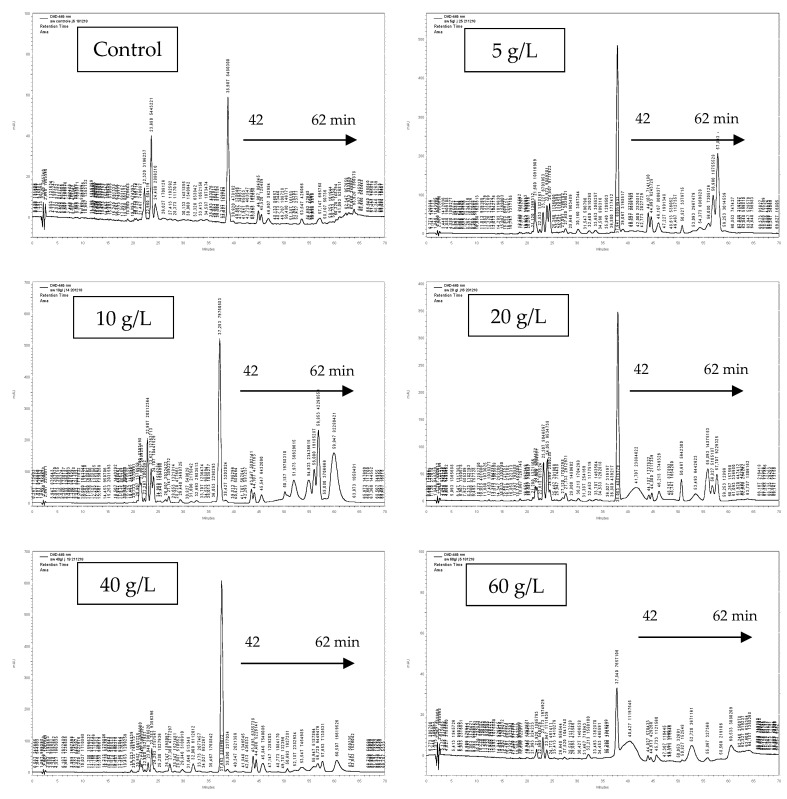
HPLC profiles of pigments detected in the control and the stressed *Scenedesmus sp.* Control: *Scenedesmus sp.* cultured in MDM medium without salt. The stressed *Scenedesmus sp.* was cultured under 5, 10, 20, 40, and 60 g/L of NaCl concentrations.

The pigment content of each extract result from 60 mg of dried biomass was measured spectrophotometrically and analyzed by analytical HPLC. Table 1 shows that *Scenedesmus* sp. stressed by 10 g/L of NaCl (Ss10) exhibited the highest content of chlorophyll a (Ca) (25.03 ± 0.03), chlorophyll b (Cb) (42.55 ± 0.63), and total carotenoid (C_carot_) (3295.84 ± 5.750) expressed in µg/mL of biomass extract solution, compared to the control and the other stressed culture extracts. In contrast, our results demonstrated that Ca and Cb contents in *Scenedesmus* sp. decrease as the salt concentrations increase from 20 to 60 g/L of NaCl, compared to the control and the 5 and 10 g/L of stressed cultures (Table 1). Moreover, Figure 2 shows that the HPLC profile of *Scenedesmus* sp. stressed by 10 g/L of NaCl presents a different pigment composition compared to the control and the other stressed culture extracts, observed from 42 to 62 min. These results could lead to the fact that 10 g/L of NaCl induces the production of new pigments by *Scenedesmus* sp., as a kind of salt stress adaptation.

### 3.3. Pigment Identification

Identification of carotenoids present in Ss10 extract was performed using HPLC–MS. Data presented in Table 2 show that our extract is rich in xanthophylls eluted from 22 to 43 min, namely (di-Z)-violaxanthin, (9-Z)-neoxanthin, zeaxanthin 5,6:5’,8’-diepoxide-a, β-cryptoxanthin 5,6-5’,6’-diepoxide, β-cryptoxanthin 5’,6’-epoxide, (all-*E*)-zeinoxanthine, (all-*E*)-violaxanthin, (all-*E*)-luteoxanthin, (9-*Z*)-antheroxanthine, lutein, and (all-*E*) zeaxanthin. The extract also contains carotene and chlorophyll pigments. The major identified pigment in Ss10 was the lutein with R_t_ of 42.07 min, [M + H]^+^ (m/z) 569, and λ _max_ (nm) 230/445/473 (Figure 2 and Table 2).

### 3.4. Antioxidant Activities

The high content of chlorophyll a and b and carotenoids in the Ss10 extract may contribute to a high antioxidant activity and other biological activities. Our results showed that the IC_50_ value of DPPH scavenging activity of the Ss10 extract is equal to 0.727 mg/mL, which is higher than that found in the control culture (IC_50_ = 1.55 mg/mL) (Table 3), but lower to that observed for the positive control ascorbic acid (IC_50_ = 0.013 mg/mL). Interestingly, the Ss10 extract exhibited a higher chelating capacity (FRAP) with an IC_50_ value of 0.269 mg/mL, compared to the control culture (IC_50_ = 1.163 mg/mL) (Table 3) and the positive control ascorbic acid (IC_50_ = 0.369 mg/mL).

### 3.5. Enzymatic Activities

#### 3.5.1. Tyrosinase Inhibitory Activity

The inhibitory effect of salt-stressed *Scenedesmus* sp. (Ss10) was assessed on the tyrosinase enzyme. Our results showed that both the control and the Ss10 extracts of *Scenedesmus* sp. inhibited tyrosinase activity (Table 3). The IC_50_ value of the Ss10 extract (0.698 mg/mL) is higher than that of the control extract (1.405 mg/mL). The kojic acid standard has an IC_50_ value equal to 1.989 mg/mL, which is much lower than that found in the Ss10 extract. Therefore, salt-stressed *Scenedesmus* sp. (Ss10) tested in this study may serve as an alternative material to obtain safer tyrosinase inhibitors.

#### 3.5.2. Elastase Inhibitory Activity

Salt-stressed *Scenedesmus* sp. (Ss10) and the control extracts were examined as a natural product for their anti-elastase potential. In fact, the Ss10 extract exhibits a higher amount of elastase inhibition (IC_50_ = 0.715 mg/mL) compared to the control extract (0.917 mg/mL) and to the quercetin used as a positive control (IC_50_ = 62.964 mg/mL) (Table 3). Considering our results of anti-elastase, salt-stressed *Scenedesmus* sp. extract can be considered as a good natural product for skin anti-aging.

### 3.6. Cytotoxic Activity

**Figure 3:**
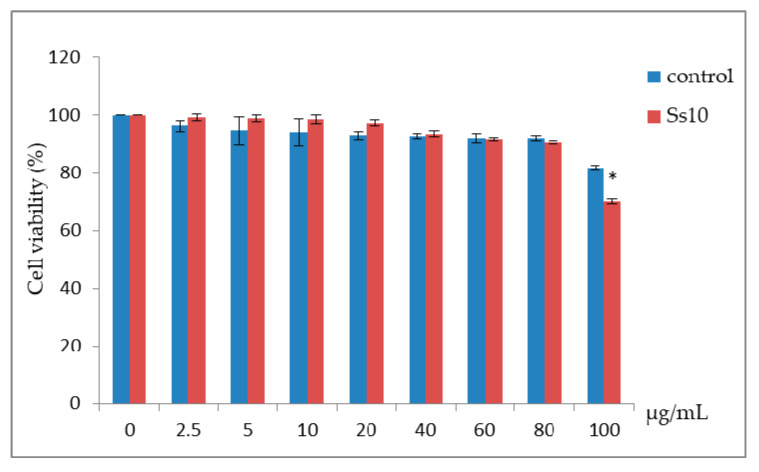
Effect of *Scenedesmus sp.* extracts on B16 cell viability. Control: *Scenedesmus sp.* cultured in MDM medium without salt. Ss10: *Scenedesmus sp.* stressed by 10 g/L of NaCl. * *p* < 0.05, significant differences, Ss10 compared with the control.

The anti-proliferative effect of the control and the Ss10 extracts was evaluated against skin melanoma cells (B16) at several concentrations (2.5–100 µg/mL). Results showed that, for concentrations lower than 100 µg/mL of both extracts, cell viability percentages were greater than 80% (Figure 3). In fact, at 100 μg/mL, the Ss10 extract reduced significantly the cell viability to 70.12%, compared to the control extract (81.72% of cell viability). In accordance with ISO 10993-5, 2009, the Ss10 extract displayed a cytotoxic effect on B16 cells at this concentration.

### 3.7. Fatty Acid (FA) Composition of Stressed Scenedesmus sp.

**Figure 4:**
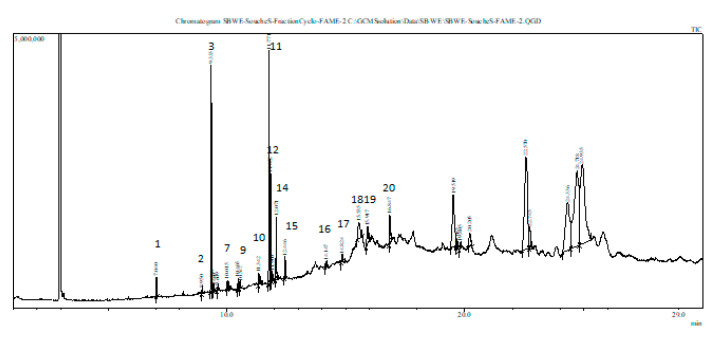
GC–MS profile of the salt-stressed *Scenedesmus* sp. (Ss10) extract. The peak assignments are listed in Table 4.

The extract of *Scenedesmus* sp. stressed by 10 g/L of NaCl contains saturated and unsaturated fatty acid (FA) compounds (Table 4). The unsaturated FAs detected (from peak 12 to 20, see Figure 4) were as follows: elaidic acid (C18:1 (trans-9)), linoleic acid (C18:2 (all cis-9,12)), palmitoleic acid (C16:1 (cis-9)), and oleic acid (C18:1 (cis-9)). Moreover, a polyunsaturated fatty acid (PUFA) (peak 15) from the omega-3 (n-3) series, namely alpha-linolenic acid (C18:3 (all cis-9,12,15)) was detected. Actually, the strain cultivated under mild salt stress showed the presence of a new unsaturated FA, namely elaidic acid (C18:1 (trans-9)) (Table 4). Those results suggest a possible commercial exploitation of the stressed strain (Ss10) in the pharmaceutical and nutritional fields.

## 4. Discussion

The effect of salt stress on *Scenedesmus* sp. growth was examined daily by measuring the OD of the cultures at 760 nm. Cultivation under salt stress was performed as follows: when the preculture of *Scenedesmus* sp. reached the exponential phase of growth, the biomass was harvested, concentrated, and transferred equally into a fresh MDM medium supplemented with 5, 10, 20, 40, or 60 g/L of NaCl. Our results showed that salt affects *Scenedesmus* sp. growth. In fact, at high salt concentrations of 40 and 60 g/L, the growth of *Scenedesmus* sp. declined gradually. These findings are in agreement with results found by Affenzeller et al. 2009 [49], who demonstrate that a high limit of salt concentration induces programmed cell death (PCD) in normal cell growth and development. Actually, the decrease in the growth causes a decrease in the biomass productivity, which presents a serious concern regarding the cultivation of microalgae under stress conditions [50]. On the contrary, our results confirmed that *Scenedesmus* sp. can tolerate and grows under a certain range of salt concentrations (from 5 to 20 g/L of NaCl). In fact, the Na^+^/H^+^ antiporters, produced by microalgae under salt stress, play an important role in the tolerance of the photosynthetic machinery [51]. Moreover, in freshwater species like *Chlamydomonas reinhardtii*, high amounts of glycerol were produced as a response to osmotic stress [14].

To determine the best salt concentration inducing the production of carotenoids by *Scenedesmus* sp., an extraction step was performed on 60 mg freeze-dried biomass of salt-stressed and control cultures. Then, the pigment contents of the extracts were measured spectrophotometrically and analyzed by analytical HPLC. Our results demonstrated that *Scenedesmus* sp. stressed by 10 g/L of NaCl (Ss10) exhibited the highest content in pigments (Table 1). Actually, the pigment content of the Ss10 extract was higher than that found in *Scenedesmus quadricauda* stressed by different salt concentrations [52], in *Scenedesmus obliquus* [53], and in other microalgae species [54]. In fact, the increase in pigment content in Ss10 may be due to the strain adaptation to the salt stress conditions, as described in previous research [22,55]. Otherwise, the pigment content of stressed *Scenedesmus* sp. decreased at high salt concentrations (Table 1). As described by Moradi and Ismail 2007 [56], the reduction in chlorophyll content at high salinities is owing to a decrease in the photosynthesis rate, caused by the osmotic stress and the toxic ionic stress of the salt. It was also demonstrated that chlorophyll is the primary target of the salt toxicity, and induces reduced photosynthesis and growth [57,58,59]. Moreover, the HPLC profile of the Ss10 extract presents a different pigment composition compared to that of the other tested extracts, observed from 42 to 62 min (Figure 2). These results could lead to the fact that 10 g/L of NaCl presents a good stress factor for *Scenedesmus* sp. to induce the production of new pigments as a kind of salt adaptation. According to Lemoine and Schoefs 2010 [60], variation in culture settings presents a stress factor to the cells, which induces the synthesis and the accumulation of carotenoids. Moreover, Kobayashi 2003 [61] demonstrated that carotenogenesis is enhanced by reactive oxygen species (ROS) generated by stress conditions like salt stress. Previous research has shown that *Scenedesmus* sp. has a carotenoid composition, presented mainly in lutein, astaxanthin, and β-carotene [53], which is responsible for the biological activities of the strain [54]. Accordingly, the higher contents of chlorophyll a and b as well as carotenoids in Ss10 may contribute to a higher antioxidant activity and other biological activities. Thus, it was selected to be chemically analyzed.

HPLC–MS has been commonly used for carotenoid identification, and mass spectra have been mostly recorded in positive ionization mode [62,63]. The ionization technique and the composition of the used mobile phase influence the fragments’ pattern observed in the mass spectra of the pigment [64]. In this study, identification of carotenoids present in the Ss10 extract was carried out using HPLC–MS, by comparing our data of UV–VIS absorption maxima (λ _max_) and molecular ions of each LC peak (MI) with other publications [65,66]. Our data showed that the Ss10 extract was rich in xanthophylls eluted from 22 to 43 min, and also contained carotene and chlorophyll pigments (Table 2). The major identified pigment in the Ss10 extract was the lutein. These findings are in agreement with those by Erdoğa et al. 2015 [65]. Additionally, Sánchez et al. 2008 [28] demonstrated that *Scenedesmus almeriensis* stressed with 5 g/L of NaCl presented a high content of lutein in its biomass composition, as a kind of adaptation to the cultivation conditions.

The antioxidant activities of DPPH radical scavenging and Fe^3+^ chelating of the Ss10 extract were higher than those found in the control culture (unstressed strain). Actually, the DPPH scavenging activity of our extract is higher than that found in *Scenedesmus quadricauda*, *Scenedesmus obliquus*, and *Tetraselmis tetrathele* [67,68,69]. Moreover, the Ss10 extract exhibited a higher ability to chelate Fe^3+^ compared to the strain tested by Custódio et al. 2014 [68]. Our findings suggest that the carotenoid compounds present in *Scenedesmus* sp. cultivated under salt stress conditions contribute significantly to the antioxidant properties. In fact, this activity was reported in some previous studies [70,71,72]. It has been demonstrated that pigments such as astaxanthin, lutein, zeaxanthin, β-carotene, and neoxanthin have a scavenging property stronger than α-tocopherol [72], while astaxanthin has the highest effect among all carotenoids [73].

In the last decade, the control of hyperpigmentation using safe inhibitors from natural sources has been given much importance. Therefore, the inhibitory effect of the Ss10 extract was assessed on the tyrosinase enzyme, which is the key enzyme of melanogenesis. The Ss10 extract of *Scenedesmus* sp. has a tyrosinase inhibition activity (Table 3), which is higher than that of the control extract (unstressed strain) and of that found in previous research [69]. This result highlights the high tyrosinase inhibition of our extract, and this may be due to the presence of carotenoids produced under salt stress conditions. Actually, the kojic acid standard, a well-known and commercial tyrosinase inhibitor, was used to make our results more meaningful. Its tyrosinase inhibition is much lower than that found in our Ss10 extract. In a previous study, Sahin 2019 [69] mentioned that the use of kojic acid has been banned in many countries because of its undesirable side effects. Therefore, mild salt-stressed *Scenedesmus* sp. tested in this study (Ss10) may serve as an alternative material to obtain safer tyrosinase inhibitors. There are only a few studies in the scientific literature that evaluate the inhibitory effect of microalgae species on the tyrosinase enzyme [74,75].

Human beings are always aspiring to look younger by using natural products. In this study, the Ss10 and control extracts of *Scenedesmus* sp. were examined as a natural product for their anti-elastase potential. Indeed, the Ss10 extract has a higher elastase inhibition activity than the control extract (unstressed strain) and the Quercetin (Table 3). The potential of elastase inhibition of our extracts is close to that found by Shirzad et al. 2018 [76]. In fact, a previous study showed that algae extract can reduce the skin extracellular matrix’s degradation by inhibiting collagenase, elastase, and metalloproteinases enzymes [76,77]. Elastase and collagenase enzymes are inhibited after inactivation of their active sites of the enzyme or after chemical modification of their substrate, by their appropriate inhibitors [78]. Considering our results of anti-tyrosinase, anti-elastase, and antioxidant activities, mild salt-stressed *Scenedesmus* sp. extract can be considered as a good natural product against hyperpigmentation, skin anti-aging, tissue engineering, and skin regeneration. For this purpose, *Scenedesmus* sp. biomass production could be produced at a larger scale in photobioreactors using the synthetic medium described in this study supplemented by 10 g/L NaCl. To save freshwater resources during microalgae production, further developments could be dedicated to the use of brackish groundwater resources (8 g/L salinity) and even the use of brine generated from brackish water desalination (12 g/L salinity).

The anti-proliferative effect of the control (unstressed strain) and the Ss10 extracts was evaluated against skin melanoma cells (B16) at several concentrations. Following ISO 10993-5, 2009, cell viability greater than 80% can be considered as non-toxic (ISO reports, 2009). Therefore, all tested concentrations lower than 100 µg/mL of both extracts are non-toxic. However, at this concentration (100 µg/mL), the Ss10 extract significantly reduced the cell viability compared to the control extract. It displayed a cytotoxic effect on B16 cells. Compared with other microalgae strains, Ss10 was efficient because of its low cytotoxic effect (no cytotoxicity below 100 µg/mL). In this context, Custódio et al. 2014 [68] reported that microalgae generally displayed a small cytotoxic effect, and found that the highest cytotoxic effect against human hepatic carcinoma HepG2 cells was achieved with hexane extracts of *Tetraselmis* sp. (IC_50_ = 58.25 μg/mL) and *Scenedesmus* sp. (IC_50_ = 93.17 μg/mL).

*Scenedesmus* sp. stressed by 10 g/L of NaCl contains saturated and unsaturated fatty acid (FA) compounds (Table 4). The fatty acid methyl ester (FAME) composition found in this study corroborates with previously published results [68,79], with some modifications. Actually, our strain cultivated under mild salt stress conditions showed the presence of linoleic acid (18:2 omega-6) and α-linolenic acid (18:3 omega3), which present the essential fatty acids for humans and animals, and a starting point for building longer chains of FAs [16]. Moreover, a new unsaturated FA, namely elaidic acid (C18:1 (trans-9)), was detected in the Ss10 extract. Therefore, those results suggest a potential commercial exploitation of the Ss10 extract in the pharmaceutical and nutritional industries. Currently, PUFAs are used especially in the aquaculture industry, considering that omega-3 PUFAs are needed to ensure the good development of fish larvae [80]. Most importantly, they are being considered as sources of omega-3 and omega-6 PUFA micronutrients for human nutrition normally found in fish and vegetable oils, respectively [81].

## 5. Conclusions

This paper’s aim was to assess the impact of salt stress on the productivity of secondary metabolites by *Scenedesmus* sp. Thus, different NaCl concentrations were tested to track changes in the growth and in pigment content of *Scenedesmus* sp. Our results showed an increase in the growth of *Scenedesmus* sp. cultured at 5, 10, and 20 g/L of NaCl concentrations and a decrease at 40 and 60 g/L of NaCl concentrations, compared to the control culture (unstressed strain). Moreover, the total chlorophyll and carotenoid contents of our strain stressed by 10 g/L of NaCl (Ss10) were higher than that found in the control and the other salt-stressed cultures. Therefore, it was selected to be chemically and biologically evaluated. HPLC–MS analysis indicated that the Ss10 extract had a rich composition in pigments, especially in xanthophylls. Furthermore, it exhibited high DPPH and FRAP antioxidant activities, as well as high tyrosinase and elastase enzymatic inhibition activities. The cytotoxicity test on B16 cells showed that the Ss10 extract was non-toxic for all tested concentrations below 100 µg/mL. Moreover, the Ss10 extract presented a rich fatty acid composition, especially in unsaturated FAs. These findings indicate that *Scenedesmus* sp. adapts to mild salt stress by the accumulation of secondary metabolites, which lead to high antioxidant and enzymatic activities. These metabolites have varied applications in health food for human consumption, aquaculture, animal feed, coloring agents, and cosmetics.

## Figures and Tables

**Table 1:** Chlorophyll a (Ca), chlorophyll b (Cb), and carotenoid (Ccarot) contents of *Scenedesmus sp.* extract solution cultured under different salt concentrations (g/L). Control: *Scenedesmus sp.* cultured in Modified Detmer’s Medium (MDM) without salt. The processed microalgae biomass was 60 mg dry weight.

Extracts	Pigments (µg/mL of Biomass Extract Solution)		
	Ca	Cb	Ccarot
Control	23.78 ± 0.87	40.49 ± 1.49	2997.45 ± 2.01
5 g/L	23.96 ± 0.53	40.83 ± 0.59	2875.31 ± 25.92
10 g/L	25.03 ± 0.03	42.55 ± 0.63	3295.84 ± 5.750
20 g/L	23.36 ± 0.82	39.73 ± 0.59	3077.67 ± 11.36
40 g/L	21.33 ± 0.02	36.27 ± 0.04	2838.11 ± 26.25
60 g/L	6.73 ± 1.18	11.47 ± 1.21	2096.96 ± 31.95

**Table 2:** Peak assignment of *Scenedesmus sp.* extract stressed by 10 g/L of NaCl (Ss10), acquired by HPLC–MS.

R_t_ (min)	[M + H]^+^ (m/z)	[M-H]^-^(m/z)	λ _max_ (nm)	Identification
22.78	601	599	406/430/457	(di-*Z*)-violaxanthin
23.75	601	599	202/440/467	(9-*Z*)-neoxanthin
24.3	601	599	402/423/449	zeaxanthin 5,6:5’,8’-diepoxide-a
24.87	585	583	432/435/461	β-cryptoxanthin 5,6-5’,6’-diepoxide
25.78	537	535	269/445/472	β-cryptoxanthin 5’,6’-epoxide
26.83	553	551	413/436/465	(all-*E*)-zeinoxanthine
27.85	601	599	416/440/469	(all-*E*)-violaxanthin
33.29	601	599	398/422/448	(all-*E*)-luteoxanthin
36.47	585	583	439/441/469	(9-*Z*)-antheroxanthine
42.07	569	567	230/445/473	lutein
43.22	568	566	224/450	(all-*E*)-Zeaxanthin
48.41	553	551	419/442/469	5,6 epoxy-α-carotene suspected
53.5	893	891	385/430/664	chlorophyll a

**Table 3:** Antioxidant and enzymatic activities of *Scenedesmus sp.* extracts. Control: *Scenedesmus sp.* cultured in MDM medium without salt. Ss10: *Scenedesmus sp.* stressed by 10 g/L of NaCl. Ascorbic acid, kojic acid, and quercetin standards are used as positive controls for antioxidant, tyrosinase, and elastase activities, respectively.

Samples	Antioxidant Activities (IC_50_, mg/mL)		Enzymatic Activities (IC_50_, mg/mL)	
Activities	DPPH	FRAP	Tyrosinase	Elastase
Control	1.550	1.163	1.405	0.917
Ss10	0.727	0.269	0.698	0.715
Ascorbic acid	0.013	0.369	-	-
Kojic acid	-	-	1.989	-
Quercetin	-	-	-	62.964

**Table 4:** Fatty acid composition of FAMEs of salt-stressed *Scenedesmus* sp. (Ss10) acquired by GC–MS.

Peaks	R_t_ (min)	Fatty Acid	Empirical Formula	Identification
1	7.040	C14:0	C_15_H_30_O_2_	Myristic acid methyl ester
3	9.333	C16:0	C_16_H_32_O_2_	Palmitic acid
7	10.012		C_17_H_26_O_2_	Methyl 4,7,10,13-hexaoctatetraenoate
10	11.342		C_19_H_40_O	n-Nonadecanol-1
11	11.771	C18:0	C_19_H_38_O_2_	Stearic acid methyl ester
12	11.832	C18:1(trans-9)	C_18_H_34_O_2_	Elaidic acid
14	12.071	C18:2 (all cis-9,12)	C_18_H_32_O_2_	Linoleic acid
15	12.446	C18:3 (all cis-9,12,15)	C_18_H_30_O_2_	alpha-Linolenic acid
18	15.535	C16:1 (cis-9)	C_16_H_30_O_2_	Palmitoleic acid
20	16.847	C18:1 (cis-9)	C_18_H_34_O_2_	Oleic acid
